# The Institutional Library

**Published:** 1913-02-15

**Authors:** 


### The Hospital

The Workers' Newspaper of Administrative Medicine and Institutional Life, Administration, National Insurance and Health.

No. 1389, Vol. LIII. SATURDAY, FEBRUARY 15, 1913. PRICE ONE PENNY.

### THE INSTITUTIONAL LIBRARY.

The often-quoted Baconian aphorism anent reading is too well known to our readers for us to cite it as an argument in favour of that study of literature, expert or otherwise, which it is in the interest of every institutional worker to cultivate. Whoever is concerned with the modern hospital, be he administrative official or member of the medical staff, knows full well that it is imperatively necessary for him to keep abreast of the times. Actual experience and frequent observation must, of course, remain the best methods of holding touch with recent improvements, new methods of construction, of treatment, and of management. But the opportunities for such observation and experience are relatively few, especially for the administrative worker, whose chances of a " study voyage " are limited to the short holidays he can manage to obtain. This being the case, we must turn to other methods and find another means of satisfying that reasonable curiosity which should animate everyone who desires to be truly progressive. We find one such means in the close study of the literature.

That is the main reason why we urge the establishment of an institutional library, though it is not the only one. One of the chief objects of such a collection is to serve for purposes of reference. There is no necessity for the library to be expensive and voluminous; a few dozen carefully selected books, so long as they are up to date and modern, will enable the worker to gain an insight into the literature far more easily and effectively than a large number ill-assorted and badly chosen. The need for referring to what has been done in or written about certain departments and certain methods arises frequently, and the worker should have at hand the means wherewith to satisfy that need. The secretary or committeeman should be able to know where he can find the statistics that he wants, where he can lay his hand on arguments or suggestions that may prove helpful to him in a discussion. The hospital architect must know where he can find certain instructive plans, handily compressed or given in detail as the case may be. Similarly the medical man connected with a hospital must have the means of finding out what has been done in his special department in relation to the general work of the institution to which he is attached. For example, a medical man may desire to know what is the relative cost of treating consumption patients and ordinary medical in-patients; his medical records will not give him that information, but a glance at the carefully compiled tables in an expert institutional handbook, such as Grotjahn, will enable him to draw a comparison at once.

Similarly, a hospital secretary may desire to know what are the medical reasons for the decline in the consumption of alcohol in hospitals. To acquire that knowledge without expert assistance means a laborious struggle through medical literature or, at the least, a perusal of special articles in certain medical papers; a brief study of a special chapter in an institutional work will give him the main points responsible for this change in such a form that he can easily grasp and understand them. New methods of treatment especially demand, so far as the lay worker is concerned, epitome and condensation, which are not usually features of medical articles. It would be impossible for a committeeman or a secretary to get an adequate conception of the pros and cons of salvarsan treatment from the medical records alone; he wants a guide and an interpreter who can make clear certain points which are obscure to him, however luminous they may be to his professional colleague. Such interpretation and guidance are afforded by the institutional journals and monographs which deal with new methods of treatment and cure mainly from the institutional point of view, and lay stress on certain facts which the purely medical writer overlooks or disregards. It may be argued that the line of demarcation between lay worker and professional is too definite to permit of interference or an attempt to coalesce or even to approach one another. That, in our opinion, is a bad argument. It is clear that the final authority on purely expert medical points must remain the expert medical man, but a difference of opinion is never likely to arise. What may, and frequently does, arise is a misconception of the relations between treatment on the one and administration on the other hand. Mutual discussion will do much to clear up such difficulties. For such mutual understanding alone do we plead. We suggest that the institutional library should be made a common ground, where the first essential kept in view is the relation between the two great interests that rule every hospital—the interests of the management and those of the medical staff. We do not suggest that these interests clash, but we do contend that they sometimes fail to correspond in that degree which, in the interest of the institution, such correspondence should reach.

In the special papers which we publish in this number of The Hospital we have attempted to outline some of the books which the institutional worker will find it to his advantage to possess in the library. The list is by no means complete, nor is it a list which cannot be revised and condensed. Once, again, let us urge the essential point that a few well selected books are worth more than a mass of volumes never read and never referred to. Obviously the most important reference books are those which give statistical evidence and plans. With such the library should be well supplied. The further choice is a matter for individual judgment, and the selection will largely depend on the taste and predilections of whoever is in charge of the collection. But the main thing after all is that the institution should possess a library, no matter how small or how incomplete. If every hospital secretary will make an effort to secure a nucleus of such a collection for his own institution we shall be far advanced on the road that leads to progress.

